# Supplementary material for: Varietal Authenticity Assessment of QTMJ Tea Using Non-Targeted Metabolomics and Multi-Elemental Analysis with Chemometrics
Source: Foods. 2023 Nov 13;12(22):4114. doi: 10.3390/foods12224114 (PMC10670169; doi:10.3390/foods12224114)
Supplement: Supplementary file 1 [file foods-12-04114-s001.zip › foods-2693927-supplementary.pdf]

Supplementary material

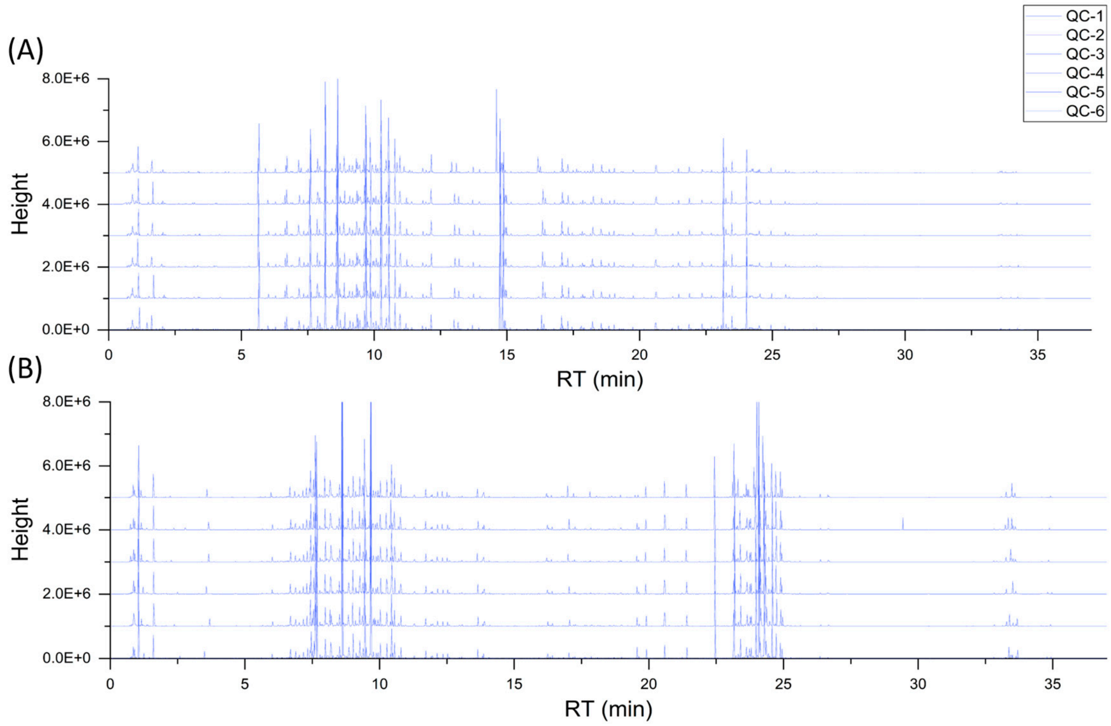

Figure S1 Total ion flow chromatograms of QC samples. (A) positive ion mode; (B) negative ion mode.

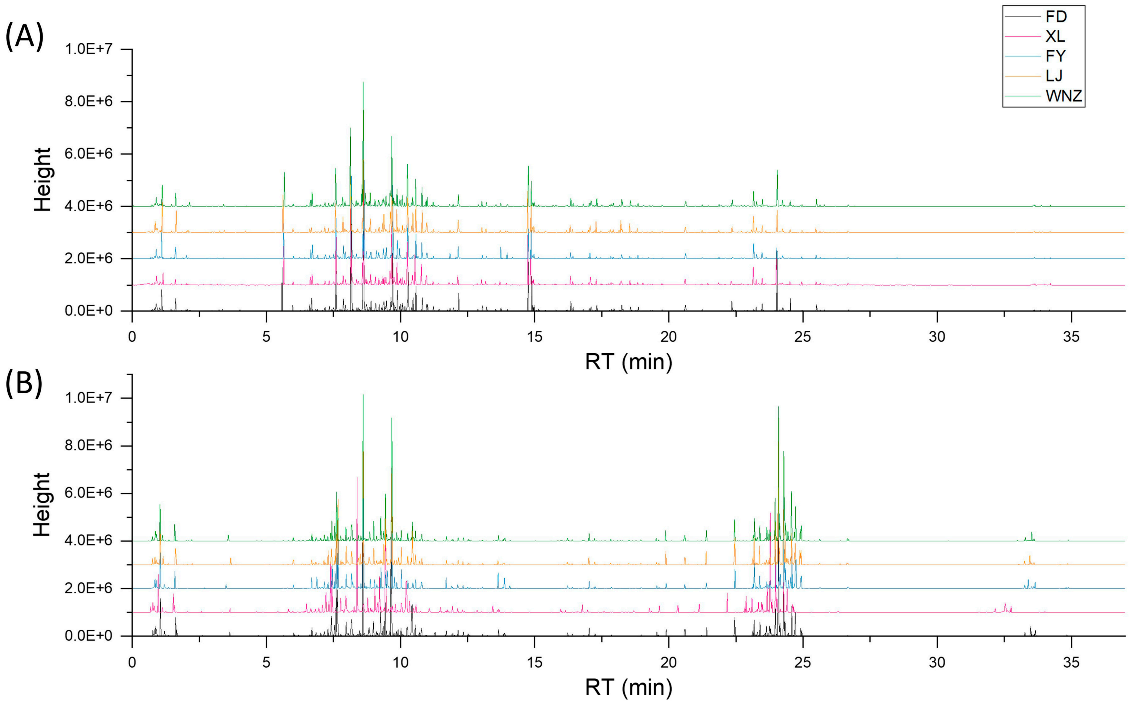

Figure S2 Total ion flow chromatograms of QTMJ tea samples from five cultivars. (A) positive ion mode; (B) negative ion mode.

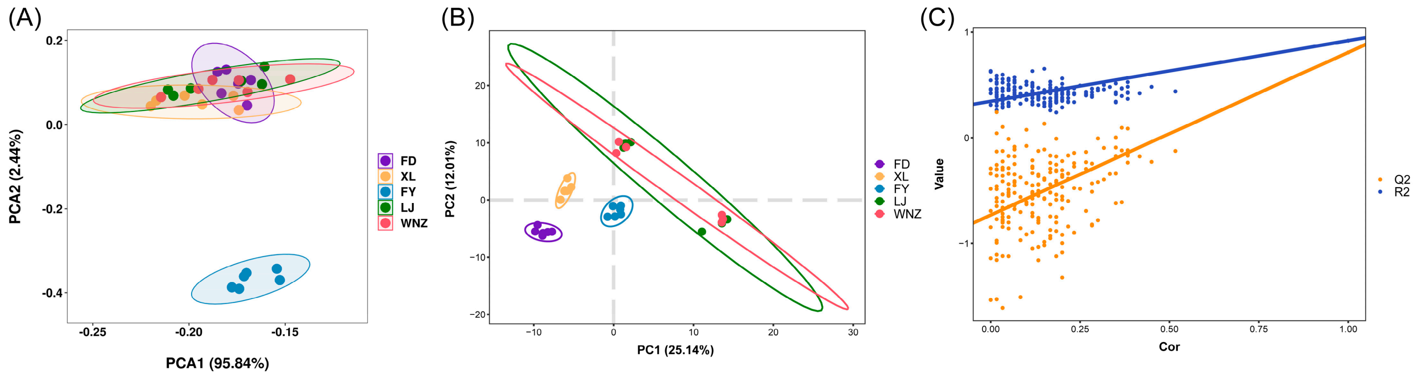

Figure S3 The discriminant results of QTMJ tea samples from five cultivars based on 442 metabolites. (A) PCA score plot; (B) PLS-DA score plot; (C) 200 permutation tests of PLS-DA.

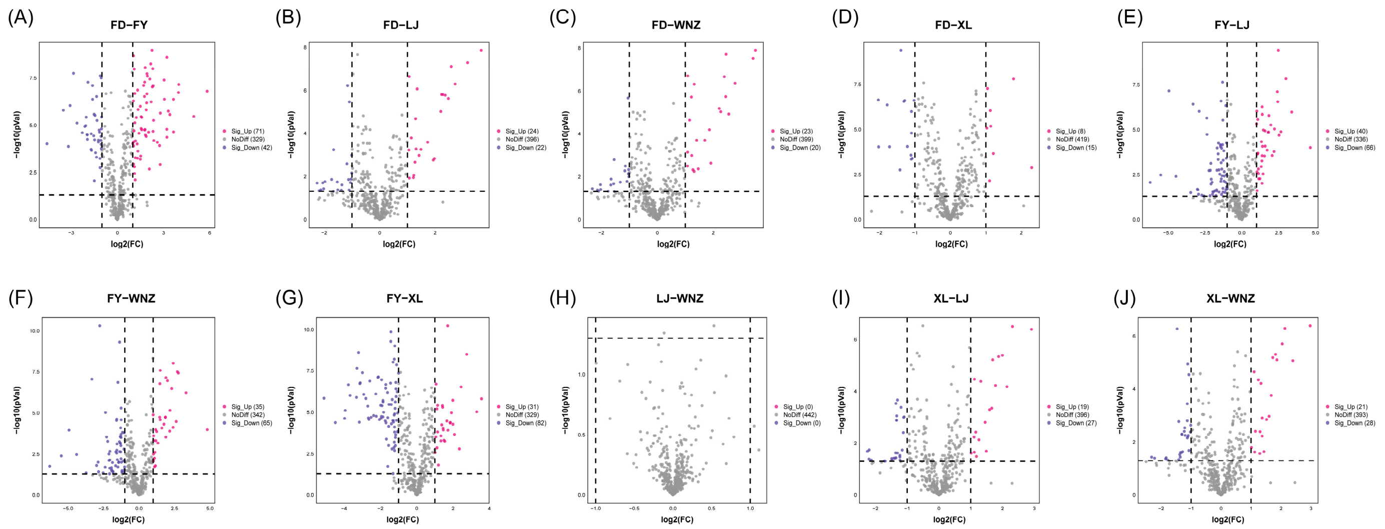

Figure S4 Volcano plots of metabolites in comparison of any pair of cultivars. (A) FD-FY; (B) FD-LJ; (C) FD-WNZ; (D) FD-XL; (E) FY-LJ; (F) FY-WNZ; (G) FY-XL; (H) LJ-WNZ; (I) XL-LJ; (I) XL-WNZ.

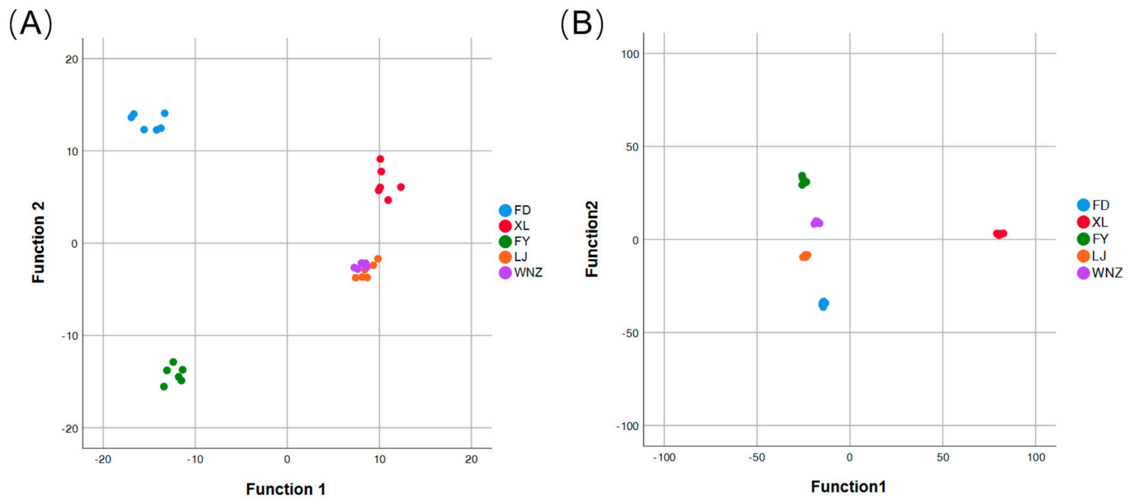

Figure S5 The discriminant results of QTMJ tea samples from five cultivars using LDA method. (A) Based on different metabolites; (B) based on mineral elements.

Table S1 The parameters for ICP-MS detection.

Parameter	Setting
RF power	1390 W
Nebulizer gas flow rate	0.90 $L \cdot min^{-1}$
Auxiliary gas flow rate	0.14 $L \cdot min^{-1}$
Coolant gas flow rate	17 $L \cdot min^{-1}$
Impact gas flow rate	3.5 $mL \cdot min^{-1}$
Sampling cone	1 mm (Ni)
Intercepting cone	0.7 mm (Ni)
Ion lens voltage	-230-40 V
Data acquisition method	Peak hopping
Number of repeated scans	5

Table S2 Sensory evaluation of different cultivars of QTMJ tea.

Cultivar	Appearance	Infusion color	Aroma	Taste	Bottom leaf	Total points
FD	94	94	94	95	93	94.2
FY	96	93	93	92	95	93.65
WNZ	95	89	86	86	90	88.95
LJ	91	90	91	89	86	89.8
XL	96	96	96	94	96	95.4

Note: The sensory evaluation method of QTMJ tea was performed according to the (GB/T 23776-2018).
